# First line therapy in stage IV BRAF mutated colorectal cancer

**DOI:** 10.1016/j.heliyon.2024.e36497

**Published:** 2024-08-22

**Authors:** Fausto Petrelli, Maria Antista, Lorenzo Dottorini, Alessandro Russo, Marcella Arru, Roberta Invernizzi, Mariangela Manzoni, Chiara Cremolini, Alberto Zaniboni, Ornella Garrone, Gianluca Tomasello, Michele Ghidini

**Affiliations:** a Oncology unit, ASST Bergamo Ovest, Treviglio, BG, Italy; b Oncology Unit, ASST Crema, Crema, CR, Italy; c Surgery Unit, ASST Bergamo Ovest, Treviglio, BG, Italy; d Oncology Unit, Fondazione Poliambulanza, Brescia, Italy; e Unit of Medical Oncology 2, Azienda Ospedaliero-Universitaria Pisana, Pisa, Italy; f Department of Translational Research and New Technologies in Medicine and Surgery, University of Pisa, Pisa, Italy; g Oncology Unit, Fondazione IRCCS Ca' Granda Ospedale Maggiore Policlinico, 20122, Milan, Italy

**Keywords:** Colorectal cancer, BRAF mutation, First-line chemotherapy, Survival, Network meta-analysis

## Abstract

**Introduction:**

The molecular profile of colorectal cancer (CRC) plays a crucial role in understanding patient prognosis and treatment response. Within CRC, a distinct subgroup can be identified by the presence of the BRAF V600E mutation. This specific mutation, classified as Class I of BRAF mutations, is known to be associated with a poor prognosis and resistance to standard therapy. To determine the most effective treatment approach for this specific subgroup of CRC, we conducted a network meta-analysis (NMA) to compare various pharmacological interventions and evaluate their relative effectiveness in BRAF-mutated CRCs.

**Materials and methods:**

On July 31, 2023, we conducted a systematic search of PubMed, Cochrane Central Register of Controlled Trials, and Embase. The inclusion criteria were as follows: 1) reporting of outcomes in patients with BRAF-mutated CRC who underwent first-line chemotherapy; 2) reporting of survival information as hazard ratios (HR); and 3) publication in English. The data were combined using HRs for overall and progression-free survival (OS and PFS) using random-effects models. NMA was performed under the Bayesian framework, utilizing the GeMTC package. The relative rankings of the treatments were determined using SUCRA scores.

**Results:**

A total of 16 studies were included. When compared to standard chemotherapy (CT) doublets (such as FOLFOX or FOLFIRI), none of the comparison arms demonstrated a gain in OS. CT doublet + bevacizumab did not show significant superiority over either CT doublet alone or 5FU/capecitabine + bevacizumab. FOLFOXIRI and FOLFOXIRI + bevacizumab did not show superiority over any other treatment schedule that was compared. CT doublets + bevacizumab had the highest SUCRA score (0.87), followed by single-agent fluoropyrimidines + bevacizumab (0.61), and FOLFOXIRI (0.56). Regarding PFS, no regimen was found to be superior to the combination of CT doublet plus bevacizumab. However, FOLFOXIRI + bevacizumab + atezolizumab showed a tendency towards better results (HR = 0.26, 95 % CI 0.05–1.1).

**Conclusions:**

Our review suggests that a CT doublet with bevacizumab is the most favorable option for OS. However, a reasonable alternative could be a triplet CT without bevacizumab.

## 1 Introduction

Metastatic colorectal cancer (CRC) is typically managed through systemic therapy, while surgical intervention may be considered for resectable metastatic sites or as a conversion treatment post-chemotherapy (CT). The molecular profile of CRC plays a crucial role in determining patient prognosis and predicting treatment response. A specific subset of CRC is characterized by the presence of the BRAF V600E mutation, which falls under Class I of the three classes of BRAF mutations. This mutation is associated with a poor prognosis and resistance to standard therapy. Approximately 10 % of metastatic CRC patients carry this mutation, which is usually mutually exclusive with RAS-mutant mutations. Right-sided primary tumors are more likely to exhibit BRAF mutations compared to left-sided tumors, resulting in shorter survival rates, an older age at diagnosis, increased mutations, KRAS alterations, and other oncogenic changes like PIK3CA, AKT1, RNF43, and SMAD4 [1]. The BRAF gene encodes a protein kinase involved in the MAPK pathway, and the valine 600 substitution leads to its constitutive activation. Historically, targeted therapies for BRAF-mutated CRC were limited, making it a challenging disease with a poor prognosis. However, BRAF inhibitors like encorafenib are now primarily used in later treatment lines following the findings of the BEACON trial [2]. For patients with unresectable disease or those undergoing conversion therapy, who have the wild-type RAS/BRAF-mutated MSS (microsatellite stable) subtype, the standard practice still involves CT alone or in combination with targeted therapies, excluding EGFR inhibitors. Pooled analyses suggest that adding cetuximab or panitumumab does not provide any benefits. In cases where intensive treatments are not suitable, MSS/proficient mismatch repair (pMMR) CRC patients typically receive CT + bevacizumab (doublet/triplet CT) or CT alone. However, determining the preferred strategy requires further discussion [[3], [4], [5]]. Previous studies have explored various combinations of CT and targeted therapies, yielding mixed results. For example, the TRIBE study suggested that the combination of 5-Fluorouracil/oxaliplatin/irinotecan triplet (FOLFOXIRI) with bevacizumab could be beneficial for BRAF-mutated CRC, but subsequent meta-analyses did not confirm these findings, underscoring the need for more personalized treatment approaches. Recent trials have also investigated the use of immunotherapy and targeted therapy combinations, such as the AtezoTRIBE study, which examined the addition of atezolizumab to FOLFOXIRI and bevacizumab. Although promising, these findings are still preliminary and require further investigation [6,7]. To determine the optimal treatment regimen for this specific subgroup of CRC, a network meta-analysis is being conducted. This analysis aims to compare different pharmacological interventions and evaluate their relative effectiveness in the management of BRAF-mutated CRCs. The review includes studies dedicated to BRAF-mutated CRCs or studies involving these patients and features a network meta-analysis of survival outcomes.

## 2 Materials and Methods

The study utilized the “Preferred Reporting Items for Systematic Reviews and Meta-Analyses (PRISMA) extension statement for reporting systematic reviews incorporating network meta-analyses.” Only randomized controlled phase II-III trials were included. Full-text studies published in peer-reviewed journals were considered, while retrospective studies were not eligible for inclusion. Studies were selected if they met the following criteria: 1) they included patients with stage IV CRC who were treated with first-line CT alone or in combination with targeted therapies, 2) they enrolled patients with RAS wt CRC and included those with any known BRAF-mutated, and 3) they provided data on overall survival and progression-free survival (OS and/or PFS) for RAS wt/BRAF-mutated subgroups. Maintenance studies, reviews, letters, commentaries, or Phase I studies were excluded.

A comprehensive literature review was conducted by searching databases such as EMBASE, Cochrane Central Register of Controlled Trials, and PubMed. The search strategy updated at July 31st, 2023 used the terms “colorectal cancer” OR “colon cancer” and “BRAF” and “randomized”. Two independent investigators (MA and FP) performed the initial step in the study selection process by screening the title, keywords, and abstract. Each of the two investigators retrieved the full-text studies and evaluated them according to the eligibility criteria. At the second step, the two investigators screened the full texts that were retrieved. Only individuals who met the eligibility criteria were included, and further analysis was conducted based on these studies. Discrepancies and disagreements during the selection process were resolved, and the studies were finalized by the third investigator (GT). After finalizing the full-text articles eligible for inclusion and analysis in the review, both investigators participated in the manual data extraction process. They used a pre-defined semi-structured data collection form. Information retrieved included the author, year of publication, country, type of study, treatments in the experimental and control arms, BRAF-mutated rate and type, and survival outcomes.

Both authors (MA and FP) assessed the risk of bias in the included studies using the “Cochrane Risk of Bias tool for Randomized Controlled Trials (RoB-2)." In the assessment for PFS and OS, contrast-based survival analyses were applied (it refers to a relative treatment effect between arms). Estimated differences in the log hazard ratio (HR) and the standard error were calculated using the published HR and confidence interval (CI). The relative treatment effects were presented as hazard ratio (HR) and 95 % credible interval (CrI). The primary outcome was OS. Forest plots were used to graphically illustrate study-specific and pooled overall estimates for the pairwise meta-analysis. In our Bayesian network meta-analysis, we utilized non-informative priors for all parameters. Non-informative priors were chosen to allow the data to primarily influence the posterior distributions. This approach is common in meta-analyses where prior information is limited or when we aim to minimize the influence of subjective prior beliefs on the results. Specifically, we used normal distributions with large variances for treatment effects and uniform distributions for variance parameters. The selection of a random-effects model was based on the high heterogeneity observed in the included studies. Random-effects models account for both within-study and between-study variability, providing a more realistic estimation of treatment effects when heterogeneity is present. This approach was crucial given the diverse patient populations, varying study designs, and different treatment regimens across the included studies.

The network meta-analysis was conducted using the GeMTC package (https://gemtc.drugis.org) under a bayesian framework which facilitates Bayesian inference through Markov Chain Monte Carlo (MCMC) simulations. We performed 40,000 iterations with a thinning interval of 10 to ensure adequate convergence and to reduce autocorrelation in the chains. Convergence diagnostics, such as trace plots and the Gelman-Rubin statistic, were monitored to confirm that the MCMC chains mixed well and converged appropriately. The Surface Under the Cumulative Ranking (SUCRA) scores were calculated to rank the treatments. SUCRA provides a comprehensive measure of the efficacy of each treatment relative to an ideal intervention. A higher SUCRA score indicates a higher probability that a treatment is among the best options.

## 3 Results

**Fig. 1:**
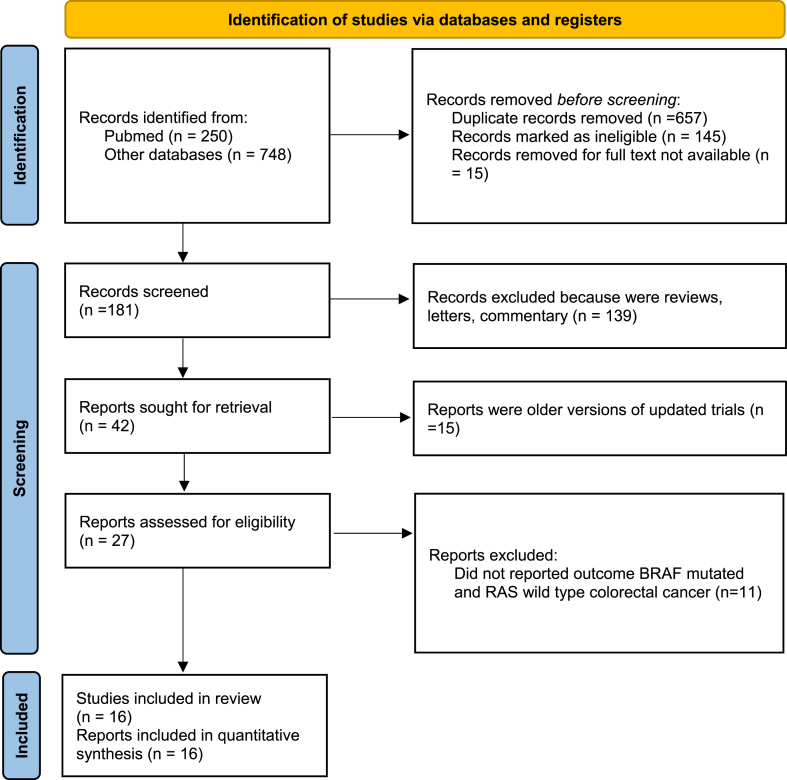
Flow diagram of included studies.

By implementing the search strategy, a total of 998 records were retrieved from all databases. After eliminating duplicate records, a primary screening of titles and abstracts was conducted on 181 records. Among these, 42 studies were deemed relevant, and full-text publications were obtained for these articles. They underwent a secondary screening based on the inclusion criteria. Ultimately, data from 16 studies that satisfied the inclusion criteria were included (Fig. 1) [[6], [7], [8], [9], [10], [11], [12], [13], [14], [15], [16], [17], [18], [19], [20], [21]]. All the included studies exhibited a low risk of bias. Table 1 presents the characteristics of the trials that were incorporated.

**Table 1:** Characteristics of included studies.

Author/years	Type of study	Median follow up (months)	Country	N° pts tot	N° pts BRAF mut experimental vs standard	Type of mutations	Experimental arm	Control arm	OS available	PFS available	Bias
Jonker 2018	Randomized, prospective	13	Canadian	103	17 (9vs8)	BRAF V600E or various	FOLFOX + bev + pelareorep	FOLFOX + bev	yes	Yes	Moderate
Modest 2019 VOLFI	Randomized phase 2	44 in experimental vs 63 in standard	Germany	96	16 (7 vs 9)	BRAF V600E or various	mFOLFOXIRI + panitumumab	mFOLFOXIRI	yes	Yes	Moderate
Bond 2023 CAIRO 5	Phase 3	51	Dutch	294	22 (12 vs 10)	BRAF V600E	FOLFOXIRI + bev	FOLFOX or FOLFIRI + bev	yes	Yes	Low
Maughan 2011 COIN	Phase 3	23	UK and Ireland	1630	102 (57 vs 45)	BRAF V600E	FOLFOX/XELOX + cetuximab	FOLFOX/XELOX	yes	Yes	Low
Innocenti 2019 CALGB 80405	Phase 3	66,5	USA	2326	100 (26 vs 41 + 33)	BRAF V600E or various	FOLFOX/FOLFIRI + bev	FOLFOX/FOLFIRI + cetuximab	yes	Yes	Low
Cremolini 2020	Meta-analisis of 5 studies	39,9	Europe and USA	1697	115 (61 vs 54)	BRAF V600E	FOLFOXIRI bev	CT doublets + bev	yes	Yes	–
Antoniotti 2022	Randomized phase 2	19,9	Italy	218	22 (12 vs 10)	BRAF V600E or various	FOLFOXIRI + bev + atezolizumab	FOLFOXIRI + bev	yes	Yes	Low
Bokemeyer 2012 CRYSTAL OPUS	Meta-analisis of 2 studies	NA	Europe	845	70 (32vs 38)	BRAF V600E or various	CT (FOLFOX/FOLFIRI) + cetuximab	CT (FOLFOX/FOLFIRI)	yes	Yes	Low
Richman 2009 FOCUS	Phase 3	NA	UK	711	56	BRAF V600E	FOLFOX/FOLFIRI	Fluoropyrimidine	yes	Yes	Low
Price 2011 AGITG MAX	Phase 3	26,5	Australia, UK New Zealand	315	33	BRAF V600E	Bev + capecitabine ± mitomycin	Capeciatnine ± mitomycin	yes	Yes	Low
Siena 2018 PRIME	Phase 3	NA	International	915	97	BRAF V600E	FOLFIRI + panitumumab	FOLFIRI	yes	Yes	Low
Rivera 2017 PEAK	Randomized phase 2	71	International	285	14(11 vs 3)	BRAF V600E or various	FOLFOX + panitumumab	FOLFOX + bev	yes	Yes	Low
Stintzing 2023 AIO KRK 0116	Randomized phase 2	NA	Germany	107	107	BRAF V600E	FOLFOXIRI + cetuximab	FOLFOXIRI + bev	yes	Yes	Moderate
Stahler 2022 AIO KRK 010	Phase 3	53	Germany	421	22 (12 VS 10)	BRAF V600E or various	Fluoropyrimidine + bev → irinotecan at PD	FOLFIRI/Capiri + bev	yes	Yes	Low
Ten hoorn 2022 CAIRO 2	Phase 3	NA	Netherlands	736	57(30 vs 27)	BRAF V600E	CAPOX + bev + cetuximab	CAPOX + bev	yes	Yes	Low

OS, overall survival; NR, not reported; CT, chemotherapy.

**Fig. 2:**
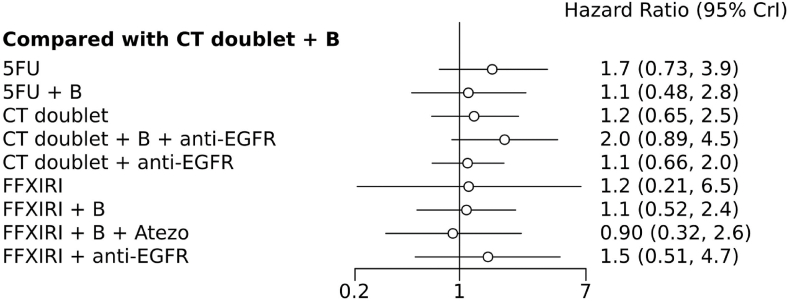
Forest plot comparing each treatment with the common comparator chemotherapy doublets + bevacizumab for the primary outcome overall survival (FFXIRI, folfoxiri; B, bevacizumab).

In comparison to standard CT doublets (such as FOLFOX or FOLFIRI), none of the comparison arms demonstrated a gain in overall survival (OS). CT doublet + bevacizumab was not significantly superior to either CT doublet alone or 5FU/capecitabine + bevacizumab. FOLFOXIRI and FOLFOXIRI + bevacizumab did not establish superiority over any other treatment schedule when compared (Fig. 2 and Suppl. Table 1). Furthermore, FOLFOXIRI + bevacizumab was similar to the triplet without bevacizumab, with a hazard ratio (HR) of 0.98 (95 % CI 0.2–4.78). Lastly, neither CT + anti-EGFR nor FOLFOXIRI + anti-EGFR exhibited superiority over any CT alone or CT + bevacizumab. The ranking of the analyzed interventions was determined using SUCRA values, which consider both the variance and magnitude of all relative intervention effects. FOLFOXIRI alone had the highest probability of being the most effective intervention (0.33), while 5FU + bevacizumab and CT doublets + bevacizumab had the highest likelihood of being the second and third best interventions. CT doublets + bevacizumab had the highest SUCRA score (0.87), followed by single-agent fluoropyrimidines + bevacizumab (0.61), and FOLFOXIRI (0.56).

**Fig. 3:**
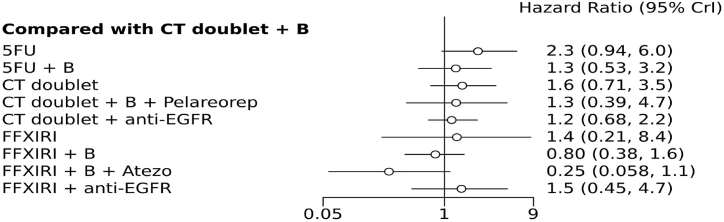
Forest plot comparing each treatment with the common comparator chemotherapy doublets + bevacizumab for the secondary outcome progression-free survival (FFXIRI, folfoxiri; B, bevacizumab).

In terms of progression-free survival (PFS), no regimen was found to be superior to the combination of CT doublet plus bevacizumab (Fig. 3 and Suppl. Table 2). However, FOLFOXIRI + bevacizumab + atezolizumab demonstrated a trend towards better results (HR = 0.26, 95 % CI 0.05–1.1). This last regimen was significantly superior to CT + anti-EGFR, CT doublets, and CT monotherapy. FOLFOXIRI + bevacizumab + atezolizumab had the highest probability of being the most effective intervention, with an 88 % probability. It also had the highest SUCRA score (0.98), followed by the CT doublet + bevacizumab combination (0.82).

**Fig. 4:**
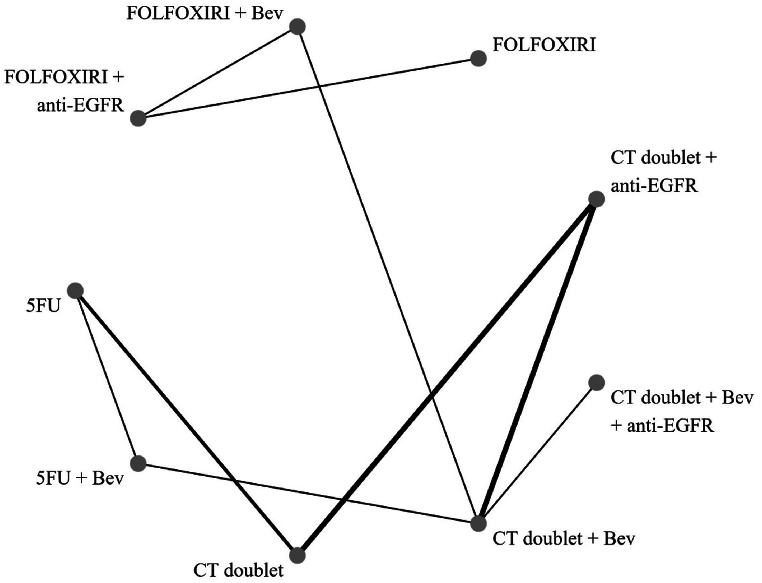
Network plot showing direct comparison of each treatment for primary outcome overall survival.

**Fig. 5:**
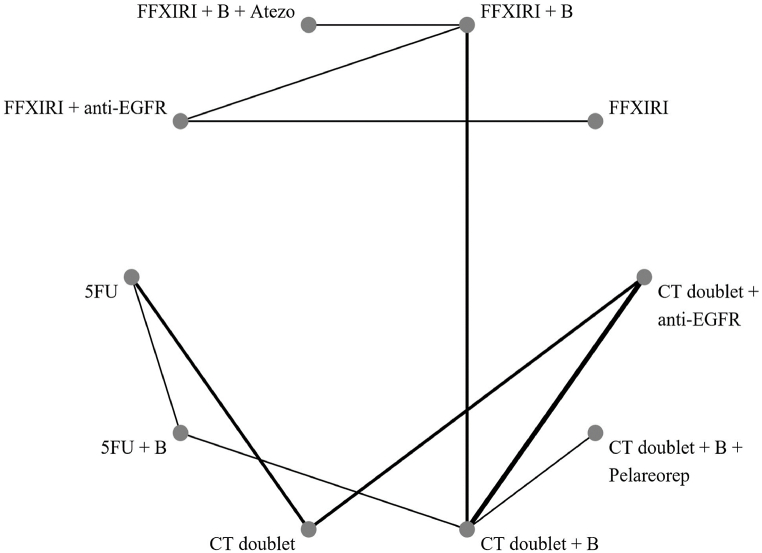
Network plot showing direct comparison of each treatment for secondary outcome progression-free survival.

The network plot (Fig. 4, Fig. 5) shows that all direct comparisons for primary endpoint.

## 4 Discussion

Treating BRAF-mutated CRC presents significant challenges. Typically, these cancers are identified at a later stage and have a poorer prognosis compared to other CRC types. Additionally, patients with these cancers do not benefit from anti-EGFR therapy. Despite the advent of targeted treatments, such as BRAF inhibitors and immune checkpoint inhibitors, conventional CT, alone or in combination with bevacizumab, remains a treatment option for patients with RAS- and BRAF-mutant CRC. However, the ideal treatment regimen is still uncertain. Treatment intensity should be tailored based on various factors, including treatment goals, patient age, performance status, and coexisting medical conditions. Our network meta-analysis indicated that in terms of OS, no CT regimen (be it single-agent, CT doublets, or FOLFOXIRI) showed a statistically significant advantage over the combination of CT and bevacizumab. The analysis also suggested that for patients who can tolerate anti-angiogenic agents, a CT doublet plus bevacizumab might be the preferred option.

Alternatively, a single-agent CT with bevacizumab or FOLFOXIRI could be considered for patients suitable for intensive therapy. Based on the SUCRA p score for PFS, CT doublet plus bevacizumab is ranked as the second-best option. Although the combination of CT + atezolizumab + bevacizumab has shown promising PFS outcomes, long-term data are lacking. This conclusion is drawn from a limited sample of 22 BRAF-mutated CRC patients in the AtezoTRIBE study, warranting cautious interpretation before considering it as a standard treatment option.

Historically, bevacizumab plus CT doublets have been used for BRAF- or RAS-mutant CRC due to the ineffectiveness of anti-EGFR agents. Studies and clinical trials have evaluated FOLFOXIRI in combination with bevacizumab, especially for right-sided and/or BRAF-mutated cancers, focusing on conversion surgery and suitable patients. This recommendation stems from a subgroup analysis of the TRIBE study's phase III, which showed a significant benefit from FOLFOXIRI + bevacizumab over FOLFIRI + bevacizumab in the BRAF-mutant subgroup. However, a subsequent meta-analysis did not confirm these initial findings, suggesting this regimen might not be preferable unless rapid tumor reduction or conversion surgery is the goal [[22], [23]].

Current trials are exploring first-line treatments with anti-BRAF agents (e.g., encorafenib), cetuximab, and standard CT. Notably, patients with BRAF V600E mutations and microsatellite unstable (MSI-H) status generally have a better prognosis, with immunotherapy being the most effective first-line treatment. Conversely, MSS CRC does not respond well to immune checkpoint inhibitors. Recent studies suggest that combining new-generation immunotherapies with targeted therapy (e.g., spartalizumab, dabrafenib, trametinib) could offer a synergistic treatment approach for MSS CRC. Previous studies have also highlighted the role of the MAPK signaling pathway, with the combination of BRAF (dabrafenib), EGFR (panitumumab), and MEK (trametinib) inhibitors showing promise in BRAF V600E mutated CRC [[24], [25], [26]].

Our review has limitations, including its adherence to the original design of randomized controlled trials without considering BRAF status for stratification. Future studies could benefit from individual patient data analysis or network meta-analysis using original datasets for more accurate results. The indirect estimates of interventions require cautious interpretation due to the limited number of cases, which affects comparison accuracy. Moreover, we compared trials with outcomes still in preliminary stages, like the AtezoTRIBE study, which only provided PFS data. Finally, while SUCRA values provide a useful measure of the relative ranking of treatments, it is important to interpret these rankings with caution, especially when the differences between treatments are not statistically significant. The rankings may reflect minor variations that are not clinically meaningful, given the uncertainty in the estimates. Future research should focus on more granular analyses, such as individual patient data meta-analyses, to provide more definitive insights.

In CRC, resistance to CT can be influenced by several factors, including mutations in the BRAF gene. The most common mutation in CRC associated with resistance is the BRAF V600E mutation. BRAF V600E mutation leads to constitutive activation of the MAPK pathway, which promotes cell proliferation and survival. This activation can render cancer cells resistant to chemotherapy agents that target DNA replication (e.g., fluoropyrimidines like 5-fluorouracil) or DNA-damaging agents (e.g., oxaliplatin). Mutant BRAF can also upregulate proteins that inhibit apoptosis (programmed cell death), thereby promoting cell survival despite CT [25,27]. The inclusion of anti-BRAF/anti-EGFR agents in therapy has transformed the standard second-line treatment for BRAF-mutant CRC and might advance to first-line treatment. However, an optimal first-line treatment for this patient group remains to be established due to poor therapy response and short PFS. Our review suggests that a CT doublet with bevacizumab is favorable for OS, but a CT triplet without bevacizumab could be a viable alternative. The AtezoTRIBE trial's preliminary findings, which assessed atezolizumab in combination with bevacizumab-based therapy, warrant cautious interpretation due to the lack of OS data. Therefore, this should not be considered the standard initial treatment for BRAF-mutant CRC.

The management of BRAF-mutated CRC highlights the broader challenge in oncology of tailoring treatments to each patient's unique genetic profile. While conventional CT, often combined with bevacizumab, remains fundamental, our findings emphasize the complexity of improving outcomes with these regimens due to the heterogeneous nature of BRAF mutations [26]. The exploration of novel treatment combinations, including targeted therapies and immunotherapies, is crucial. However, their efficacy may be influenced by factors such as the tumor microenvironment, concurrent mutations, and the patient's immune profile, necessitating a deeper understanding of these aspects for more effective personalized treatment strategies.

Our review highlights also several areas for future research. Further research into the pharmacodynamics and molecular mechanisms underlying the effectiveness of different regimens could provide deeper insights into optimizing treatment combinations. Second, given the heterogeneity of BRAF-mutated CRC, personalized treatment strategies based on genetic, molecular, and immunological profiles are essential. Studies incorporating biomarkers to predict response to specific therapies could significantly enhance treatment outcomes. Third, incorporating real-world evidence and innovative trial designs could accelerate the translation of research findings into clinical practice. Prospective clinical trials should also focus on patient-reported outcomes and quality of life measures to ensure that treatment benefits outweigh potential adverse effects. Fourth, optimizing the sequencing and combination of therapies is an ongoing area of research. The potential of the MAPK pathway as a therapeutic target in BRAF-mutated CRC is particularly intriguing, with the activity observed in combinations of BRAF, EGFR, and MEK inhibitors suggesting a viable strategy. However, the development of such combinations must consider toxicity risks and patient quality of life. Finally, our review underscores the need for well-designed prospective clinical trials to identify effective treatment combinations and understand the biological drivers of treatment response and resistance. Incorporating real-world evidence and innovative trial designs could accelerate the application of scientific discoveries in clinical settings.

In conclusion, the treatment landscape for BRAF-mutated CRC is evolving, with our review identifying bevacizumab combined with CT doublets as a favorable option for OS. Advances in targeted therapy and immunotherapy hold promise for more effective, personalized approaches. However, realizing this potential requires unraveling the complex genetic, molecular, and immunological factors influencing treatment outcomes in BRAF-mutated CRC. A multidisciplinary approach integrating clinical and research insights is essential for improving the prognosis and quality of life for patients with this challenging cancer subtype. The selection of initial therapy should consider various factors, including disease severity, treatment goals, the patient's performance status, age, and expectations.

The data supporting this study are available from the corresponding author upon reasonable request.

## CRediT authorship contribution statement

**Fausto Petrelli:** Writing – review & editing, Writing – original draft, Validation, Software, Methodology, Formal analysis, Data curation, Conceptualization. **Maria Antista:** Validation, Data curation. **Lorenzo Dottorini:** Visualization, Validation. **Alessandro Russo:** Validation. **Marcella Arru:** Validation. **Roberta Invernizzi:** Validation. **Mariangela Manzoni:** Validation. **Chiara Cremolini:** Validation. **Alberto Zaniboni:** Validation. **Ornella Garrone:** Validation. **Gianluca Tomasello:** Validation. **Michele Ghidini:** Writing – review & editing, Writing – original draft, Visualization, Validation.

## Declaration of competing interest

The authors declare that they have no known competing financial interests or personal relationships that could have appeared to influence the work reported in this paper.
