# Supplementary material for: First line therapy in stage IV BRAF mutated colorectal cancer
Source: Heliyon. 2024 Aug 22;10(17):e36497. doi: 10.1016/j.heliyon.2024.e36497 (PMC11388748; doi:10.1016/j.heliyon.2024.e36497)
Supplement: Multimedia component 1 [file mmc1.docx]

| 5FU | 0.696 ( 0.357, 1.364) | 0.758 ( 0.478, 1.210) | 0.621 ( 0.336, 1.142) | 1.251 ( 0.563, 2.764) | 0.700 ( 0.412, 1.192) | 0.708 ( 0.156, 3.122) | 0.689 ( 0.329, 1.410) | 0.558 ( 0.256, 1.194) | 0.966 ( 0.390, 2.347) |
| --- | --- | --- | --- | --- | --- | --- | --- | --- | --- |
|  | 5FU + Bev | 1.089 ( 0.539, 2.202) | 0.890 ( 0.450, 1.756) | 1.791 ( 0.769, 4.160) | 1.003 ( 0.495, 2.027) | 1.022 ( 0.223, 4.655) | 0.984 ( 0.451, 2.189) | 0.801 ( 0.354, 1.828) | 1.382 ( 0.535, 3.537) |
|  |  | CT doublet | 0.817 ( 0.509, 1.310) | 1.646 ( 0.826, 3.296) | 0.925 ( 0.694, 1.232) | 0.935 ( 0.220, 3.855) | 0.909 ( 0.494, 1.667) | 0.737 ( 0.382, 1.416) | 1.272 ( 0.574, 2.824) |
|  |  |  | CT doublet + Bev | **2.012 ( 1.215, 3.316)*** | 1.133 ( 0.764, 1.672) | 1.141 ( 0.293, 4.411) | 1.108 ( 0.747, 1.639) | 0.899 ( 0.564, 1.435) | 1.558 ( 0.809, 2.985) |
|  |  |  |  | CT doublet + Bev + anti-EGFR | 0.561 ( 0.295, 1.063) | 0.568 ( 0.134, 2.414) | 0.550 ( 0.292, 1.041) | **0.446 ( 0.225, 0.894)*** | 0.772 ( 0.337, 1.756) |
|  |  |  |  |  | CT doublet + anti-EGFR | 1.012 ( 0.244, 4.136) | 0.981 ( 0.565, 1.691) | 0.796 ( 0.433, 1.452) | 1.381 ( 0.644, 2.956) |
|  |  |  |  |  |  | FOLFOXIRI | 0.967 ( 0.268, 3.565) | 0.786 ( 0.213, 2.971) | 1.366 ( 0.425, 4.517) |
|  |  |  |  |  |  |  | FOLFOXIRI + Bev | 0.812 ( 0.630, 1.042) | 1.406 ( 0.832, 2.376) |
|  |  |  |  |  |  |  |  | FOLFOXIRI + Bev + Atezo | 1.732 ( 0.969, 3.102) |
|  |  |  |  |  |  |  |  |  | FOLFOXIRI + anti-EGFR |

**Suppl. Tab. 1 Comparison of the included interventions for OS: hazard ratio (95% CrI). Each cell gives the effect of the column-defining intervention relative to the row-defining intervention (grey are direct comparisons; orange indirect comparisons).**

| 5FU | 0.589 ( 0.25, 1.12) | 0.67 ( 0.35, 1.3) | 0.44 ( 0.17, 1.03) | 0.583 ( 0.130, 2.665) | 0.520 ( 0.231, 1.189) | 0.605 ( 0.077, 4.080) | 0.354 ( 0.108, 1.033) | **0.113 ( 0.01, 0.59)*** | 0.667 ( 0.151, 2.619) |
| --- | --- | --- | --- | --- | --- | --- | --- | --- | --- |
|  | 5FU + Bev | 1.16 ( 0.52, 2.96) | 0.75 ( 0.32, 1.87) | 1.005 ( 0.233, 4.664) | 0.895 ( 0.392, 2.392) | 1.036 ( 0.144, 7.075) | 0.605 ( 0.195, 1.884) | 0.194 ( 0.03, 1.1) | 1.142 ( 0.278, 4.747) |
|  |  | CT doublet | 0.64 ( 0.28, 1.42) | 0.856 ( 0.198, 3.633) | 0.771 ( 0.411, 1.455) | 0.890 ( 0.121, 5.794) | 0.521 ( 0.169, 1.442) | **0.16 ( 0.02, 0.85)*** | 0.991 ( 0.228, 3.741) |
|  |  |  | CT doublet + Bev | 1.325 ( 0.393, 4.512) | 1.190 ( 0.671, 2.218) | 1.363 ( 0.228, 7.731) | 0.802 ( 0.38, 1.55) | 0.25 ( 0.05, 1.07) | 1.515 ( 0.477, 4.670) |
|  |  |  |  | CT doublet + Bev + Pelareorep | 0.901 ( 0.229, 3.562) | 1.016 ( 0.122, 8.266) | 0.602 ( 0.142, 2.367) | 0.195 ( 0.02, 1.25) | 1.137 ( 0.217, 5.918) |
|  |  |  |  |  | CT doublet + anti-EGFR | 1.153 ( 0.172, 6.926) | 0.678 ( 0.249, 1.606) | **0.216 ( 0.04, 0.99)*** | 1.286 ( 0.332, 4.359) |
|  |  |  |  |  |  | FOLFOXIRI | 0.584 ( 0.119, 2.987) | 0.18 ( 0.02, 1.51) | 1.117 ( 0.286, 4.372) |
|  |  |  |  |  |  |  | FOLFOXIRI + Bev | 0.32 ( 0.08, 1.15) | 1.890 ( 0.778, 4.588) |
|  |  |  |  |  |  |  |  | FOLFOXIRI + Bev + Atezo | **5.92 ( 1.2, 28)*** |
|  |  |  |  |  |  |  |  |  | FOLFOXIRI + anti-EGFR |

**Suppl. Tab. 2 Comparison of the included interventions for PFS: hazard ratio (95% CrI). Each cell gives the effect of the column-defining intervention relative to the row-defining intervention (grey are direct comparisons; orange indirect comparisons; *:statistically significant results).**
